# Corrigendum: PasT of *Escherichia coli* sustains antibiotic tolerance and aerobic respiration as a bacterial homolog of mitochondrial Coq10

**DOI:** 10.1002/mbo3.1140

**Published:** 2020-12-22

This article corrects the following:

Cinzia Fino, Martin Vestergaard, Hanne Ingmer, Fabien Pierrel, Kenn Gerdes, Alexander Harms

## Correction regarding *Escherichia coli* O157:H7 strain EDL933

When revisiting the different enterobacterial strains used in our article (Fino et al., 2020), we discovered that *Escherichia coli* O157:H7 strain EDL933 had been misidentified. As outlined in the original article by Marinus and Poteete (2013), the strain identified as GM9255 is not a mutant of the original EDL933 strain in which the Shiga toxin genes encoded in the 933w prophage have been inactivated (which would be GM9251), but instead an *E*.* coli* K‐12 derivative that had been lysogenized with this engineered prophage. The results presented in appendix figures A3, A5, and A6 with strain GM9255—identified as “*E*.* coli* O157:H7 EDL933”—are therefore not informative about the biology of PasTI in an *E*.* coli* O157:H7 strain. Due to the close relationship of RatAB/PasTI in all organisms used in our work, we did not recognize this misidentification when constructing the Δ*pasTI* mutant of the strain that we thought was *E*.* coli* O157:H7 EDL933. This misidentification does not affect any conclusions of our work, because all data shown in the main figures have been generated with our primary model organisms *Escherichia coli* CFT073 and *Escherichia coli* K‐12 MG1655. The strain that we thought was *E*.* coli* O157:H7 EDL933 has only been indicated in appendix figures A3, A5, and A6 to demonstrate the general relevance of our findings for enterobacteria beyond our model organisms. For this purpose, the data generated with this strain were always paired with comparable data generated with pathogenic *E*.* coli* strain 55989 and *Salmonella enterica* strain SR‐11 that yielded similar results. The authors apologize for this oversight.
